# Progress and prospects in antisense oligonucleotide-mediated exon skipping therapies for Duchenne muscular dystrophy

**DOI:** 10.1007/s10974-024-09688-2

**Published:** 2025-01-30

**Authors:** Katarzyna Chwalenia, Matthew J. A. Wood, Thomas C. Roberts

**Affiliations:** 1 Institute of Developmental and Regenerative Medicine, University of Oxford, IMS-Tetsuya Nakamura Building, Old Road Campus, Roosevelt Dr, Headington, Oxford, OX3 7TY UK; 2 Department of Paediatrics, University of Oxford, South Parks Road, Oxford, OX1 3QX UK; 3 MDUK Oxford Neuromuscular Centre, Oxford, OX3 7TY UK

**Keywords:** Duchenn muscular dystrophy, DMD, Antisense oligonucleotide, Exon skipping, Dystrophin

## Abstract

Recent years have seen enormous progress in the field of advanced therapeutics for the progressive muscle wasting disease Duchenne muscular dystrophy (DMD). In particular, four antisense oligonucleotide (ASO) therapies targeting various DMD-causing mutations have achieved FDA approval, marking major milestones in the treatment of this disease. These compounds are designed to induce alternative splicing events that restore the translation reading frame of the dystrophin gene, leading to the generation of internally-deleted, but mostly functional, pseudodystrophin proteins with the potential to compensate for the genetic loss of dystrophin. However, the efficacy of these compounds is very limited, with delivery remaining a key obstacle to effective therapy. There is therefore an urgent need for improved ASO technologies with better efficacy, and with applicability to a wider range of patient mutations. Here we discuss recent developments in ASO therapies for DMD, and future prospects with a focus on ASO chemical modification and bioconjugation strategies.

## Dystrophinopathy

D﻿uch﻿enne muscular dystrophy is a severe, X-linked inherited disorder caused by loss of the dystrophin protein affecting 1:3500–5000 boys worldwide (Hoffman et al. 1987). The disease results from a spectrum of loss-of-function mutations in the dystrophin gene (*DMD*) (Hoffman et al. 1987), the most common of which are whole exon deletions (Muntoni et al. 2003). *DMD* is one of the largest genes of the human genome, encoding a 14 kb, 79-exon long mRNA which is translated into a 427 kDa dystrophin protein. In DMD, dystrophin deficiency results in vulnerability of muscles to contraction-induced damage (Petrof et al. 1993). As such, daily physical function initiates a detrimental cascade of degeneration-regeneration cycles, ultimately leading to muscle wasting (Cardone et al. 2023). Notably, the disease affects not only skeletal muscle, but also the heart, and almost all DMD patients develop a form of dilated cardiomyopathy, often leading to end-stage heart failure (Szabo et al. 2021). Currently, cardiac dysfunction is the main cause of death for dystrophic patients, typically in the third decade of life (Passamano et al. 2012).

Becker muscular dystrophy is an allelic form of DMD characterised by later onset, milder disease severity, and slower disease progression, with many patients remaining ambulant well into adulthood (with some never requiring wheelchair use) (England et al. 1990; Clemens et al. 2020a; Thada et al. 2023). In most cases, the difference between the DMD and BMD phenotypes can be explained by the ‘reading frame rule’ (Vengalil et al. 2017; Aartsma-Rus et al. 2006). Specifically, DMD is caused by frameshift mutations which lead to production of defective, prematurely terminated protein variants. Conversely, BMD patients typically maintain the translation reading frame, producing an internally deleted, yet partially functional, pseudodystrophin, which underlies the milder disease progression. A remarkable example of this phenomenon is the case of a BMD patient lacking 46% of the dystrophin gene who presented with only mild symptoms and was ambulant at the age of 61 (England et al. 1990).

## Antisense oligonucleotide therapeutics

Antisense nucleotides (ASOs) are short, single-stranded nucleic acid polymers (~ 20–30 nucleotides in length) designed to bind to complementary target mRNA sequences via Watson–Crick base pairing (Roberts et al. 2023, 2020; Aartsma-Rus et al. 2017; Brad Wan and Seth 2016). The therapeutic potential of ASOs was first demonstrated in 1978 by Stephenson and Zamecnik who showed that short, synthetic DNA oligonucleotides can inhibit replication of the Rous sarcoma virus in a cell-free system (Stephenson and Zamecnik 1978). These results have paved the way for research into short oligonucleotide-based therapeutics, and eventually to the regulatory approval of ~ 15 ASO-based drugs over the past 26 years, the vast majority within the last decade (Lauffer et al. 2024). ASOs elicit gene regulatory functions via a variety of mechanisms including post-transcriptional control through transcript degradation, translation inhibition, or splice-switching (Lauffer et al. 2024; Dhuri et al. 2020). In the context of DMD, ASO-mediated exon skipping of the dystrophin pre-mRNA is one of the most promising therapeutic strategies (Roberts et al. 2023). This approach aims to convert the severe DMD phenotype into a less severe ‘BMD-like’ phenotype by paradoxically increasing the degree of internal deletion and restoring the translation reading frame in the dystrophin protein via the exclusion of a specific exon(s) from the *DMD* pre-mRNA. This leads to the production of an internally-deleted but largely functional pseudodystrophin protein (Roberts et al. 2023; Aartsma-Rus et al. 2009). Importantly, ASO-mediated exon skipping is a mutation-specific approach, theoretically applicable to as many as ~ 83% of all DMD-causing mutations (Aartsma-Rus et al. 2009; Bladen et al. 2015).

Due to their inherent susceptibility to nucleases and poor target binding affinity, unmodified ASOs based on natural DNA and RNA chemistry have limited clinical utility (Roberts et al. 2020). As such, diverse ASO chemistries have been developed to improve their ‘drug-likeness’ (Roberts et al. 2020). The most commonly used nucleic acid chemistry in DMD exon skipping therapeutics is PMO (phosphorodiamidate morpholino oligonucleotide). PMOs consist of a six-membered morpholine ring (analogous to the five-membered arabinose ring found in native DNA/RNA) and an uncharged phosphorodiamidate backbone (analogous to the phosphodiester linkages). The non-natural chemical composition of PMOs means that they cannot be degraded by biofluid or tissue nucleases, and as such, these molecules exhibit high stability and a very favourable safety profile (Amantana and Iversen 2005; Summerton and Weller 1997). Indeed, PMOs have been administered to rodents at doses of up to 3 g/kg (Wu et al. 2010). As of 2024, four PMO antisense drugs have been approved by the FDA for treatment of DMD (Table 1): eteplirsen, golodirsen, viltolarsen, and casimersen (Lim et al. 2017; Roshmi and Yokota 2023; Clemens et al. 2020b; Heo 2020; Shirley 2021).

**Table 1:** FDA-approved ASO exon skipping therapies for DMD

ASO	*DMD* Target exon	Sequence (5ʹ to 3ʹ)	Company	FDA approval	Dystrophin protein restoration	References
Eteplirsen	51	CTCCAACATCAAGGAAGATGGCATTTCTAG	﻿Sarepta	Sept 2016	0.9% after 180 weeks	Lim et al. (2017)
Golodirsen	53	GTTGCCTCCGGTTCTGAAGGTGTTC	Sarepta	Dec 2019	1% after 48 weeks	Heo (2020; Servais et al. (2022)
Viltolarsen	53	CCTCCGGTTCTGAAGGTGTTC	NS Pharma	Aug 2020	5.9% after 25 weeks	Roshmi and Yokota (2023); Clemens et al. (2020b)
Casimersen	45	CAATGCCATCCTGGAGTTCCTG	Sarepta	Feb 2021	4.25% after 48 weeks	Shirley (2021); Nicolau et al. (2024)

All ASO sequences consist of phosphorodiamidate morpholino oligonucleotide (PMO) chemistry

Eteplirsen (developed by Sarepta Therapeutics) was the first drug to receive marketing authorisation in 2016 (Lim et al. 2017), which marked a significant milestone in the development of therapeutics for DMD (Lim et al. 2017). This was the first gene-specific drug offered to DMD patients, which utilised a novel, targeted mechanism as opposed to the pleiotropic mode of action of corticosteroids, which are the standard-of-care in DMD management (Bushby et al. 2010). The clinical trials leading to the eteplirsen approval began in 2007 with a first phase 1/2 study conducted at Imperial College London demonstrating the safety and tolerability of the drug after intramuscular injection in patients (NCT00159250) (Kinali et al. 2009). A further, phase 2, dose escalation, intravenous injection trial demonstrated a positive safety profile for eteplirsen, with no drug-related serious adverse events reported in the cohort of 19 ambulant DMD patients (NCT00844597) (Cirak et al. 2011). The exon-skipping efficacy, and thus therapeutic benefit, of the drug were variable in these studies with a maximum increase of 16% in dystrophin levels as measured by western blot in the phase 2 trial (Cirak et al. 2011). Follow-up studies reported more modest findings. Treatment with weekly injections of eteplirsen for almost 3.5 years resulted in the restoration of ~ 1% of dystrophin levels observed in healthy muscle (Lim et al. 2017). As such, the approval of eteplirsen by FDA in 2016 was highly controversial, as it was solely based on only minimal dystrophin restoration as a surrogate endpoint, without the evidence of functional benefit (Aartsma-Rus and Goemans 2019). Similarly modest results were obtained with other approved ASOs (Table 1) (Lim et al. 2017; Roshmi and Yokota 2023; Clemens et al. 2020b; Heo 2020; Shirley 2021; Servais et al. 2022; Komaki et al. 2018; Nicolau et al. 2024). For example, dystrophin restoration levels were the highest for viltolarsen, with 5.9% of dystrophin expression achieved after 25 weeks of treatment (Clemens et al. 2020b). Clinical trials for all of the FDA-approved exon skipping ASOs are currently ongoing (Vincik et al. 2024). Notably, none of the FDA-approved ASOs for DMD have been approved by European Medicine Agency, based on the same efficacy data (Aartsma-Rus and Goemans 2019).

## Enhancing ASO activity

Due to the limited efficacy of available drugs, the pursuit of improved ASO therapeutics in DMD continues. Here we will consider the two major approaches for ASO improvement; (i) chemical modification, and (ii) bioconjugation.

A major development in oligonucleotide chemistry is the incorporation of stereopure backbone linkages. While phosphodiester backbones are achiral, the incorporation of a sulphur to replace one of the non-bridging backbone oxygen atoms, as in the case of phosphorothioate modification, gives rise to chiral centres at every backbone linkage position. Control of the stereochemistry at each position has been reported to improve many oligonucleotide properties, with a stereopure enantiomer ultimately being more potent than the racemic bulk mixture (Kandasamy et al. 2022a). To this end, Wave Life Sciences developed a stereopure ASO with phosphorothioate backbone to induce *DMD* exon 51 skipping (suvodirsen) (Wagner et al. 2019). Despite promising pre-clinical results, suvodirsen failed to meet its primary endpoint (i.e., an increase in dystrophin protein expression) in a phase I clinical trial (NCT03508947), and its development was subsequently discontinued (Aartsma-Rus and Corey 2020). However, Wave Life Science have pursued a second stereopure ASO designed to skip *DMD* exon 53 (WVE-N531). This compound is a mixmer ASO containing 2ʹ-*O*-methyl and 2ʹ-fluoro sugar modifications, and a mixture of stereoselective phosphorothioate and phosphoryl guanidine (PN) backbone linkages (Kandasamy et al. 2022b). This pattern of ASO chemistry has been shown to improve delivery and overall therapeutic potency in animal models, including the severely affected dystrophin/utrophin double knock-out (dKO) mouse model of DMD (Kandasamy et al. 2022a; Tillinger et al. 2023). WVE-N531 is currently being evaluated in a phase 1b/2 trial (FORWARD-53, NCT04906460). Initial reports from this trial have been highly encouraging, with a mean level of exon skipping of 53% after three biweekly 10 mg/kg doses in the first three DMD patients treated, although dystrophin protein levels were negligible (Tillinger et al. 2023). Interim data from this trial after 24 weeks of dosing recently reported mean dystrophin protein expression levels of 9% (muscle content-adjusted) and 5.5% (unadjusted), with mean exon skipping levels of 57% (Wave life sciences announces positive interim data from FORWARD-53 Clinical Trial Evaluating WVE-N531 in boys with Duchenne muscular dystrophy amenable to exon 53 skipping - wave life sciences Available from: https://ir.wavelifesciences.com/news-releases/news-release-details/wave-life-sciences-announces-positive-interim-data-forward-53).

Notably, a 2ʹ-*O*-methyl ASO with phosphorothioate backbone, developed by BioMarin (drisapersen) was ultimately discontinued after demonstrating limited efficacy (i.e., lack of functional improvement) and adverse events including injection site reactions, thrombocytopenia, and proteinuria in a phase 3 trial (NCT01254019) (Markati et al. 2021; Goemans et al. 2018). The incorporation of phosphorothioate linkages was associated with dose limiting renal toxicity, which may prove to be a challenge for other therapeutic modalities, given the widespread usage of this chemistry in the nucleic acid therapeutic field. Nevertheless, other phosphorothioate containing ASOs have also been investigated. Daiichi Sankyo was developing renadirsen (DS 5141b) a fully phosphorothioate mixmer containing 2ʹ-*O*-methyl and ethylene bridged nucleic acid (ENA) modifications for the skipping of *DMD* exon 45 (Ito et al. 2021), investigated in a phase 2 clinical trial (NCT04433234), although development of this drug is now discontinued. Furthermore, SQY Therapeutics is investigating a completely distinct chemistry, tricyclo-DNA. An ASO consisting of this chemistry, and conjugated to a palmitic acid, (SQY51) designed to skip *DMD* exon 51 is currently under investigation in a phase 1/2 clinical trial (NCT05753462/AVANCE1). Tricyclo-DNA is notable, as it has been reported to enable exon skipping in the brain, meaning that the neurological pathologies of DMD may be treatable (Goyenvalle et al. 2015; Zarrouki et al. 2022).

A key advantage of FDA-approved PMO chemistry is the favourable safety profile resulting from their low plasma protein binding properties (Roberts et al. 2020; Brad Wan and Seth 2016), although this same characteristic also leads to rapid plasma clearance of the PMO via the kidneys (Roberts et al. 2020; Brad Wan and Seth 2016; Vincik et al. 2024; Tsoumpra et al. 2019; Betts et al. 2012). This results not only in the requirement for weekly dosing for prolonged periods, but also in limited uptake by targeted cells. As such, the efficient delivery of PMO to DMD muscle (and especially heart) remains an ongoing challenge for the field. An alternative approach for ASO improvement is via covalent conjugation to a delivery-assisting moiety. This has been most extensively studied in the case of PMOs, whereby the uncharged backbone enables facile synthesis of a variety of conjugates.

One of the most promising technologies for improved tissue targeting and uptake is conjugation of the PMO with cell-penetrating peptides (CPPs) (Tsoumpra et al. 2019; Betts et al. 2012). CPPs are short (< 30 amino acids), often cationic, sequences that possess the remarkable ability to cross cell membranes, making them valuable tools for delivering therapeutic molecules (McClorey and Banerjee 2018). A plethora of peptide-conjugated PMOs (PPMOs) have been developed and pre-clinically tested in the context of DMD (Tsoumpra et al. 2019). In comparison to unconjugated PMOs, PPMOs demonstrate enhanced efficacy as splicing correctors at lower doses, enabling systemic administration to achieve widespread dystrophin restoration in skeletal muscles and cardiac tissue (Tsoumpra et al. 2019). Multiple recent clinical trials of PPMOs for DMD are ongoing or have recently completed: Sarepta (NCT04004065/MOMENTUM, SRP-5051/vesleteplirsen), PepGen (NCT06079736, PGN-EDO51) and Entrada (ENTR-601-44-101, ENTR-601-44) (Roberts et al. 2023). The interim phase 2 data of vesleteplirsen (which consists of a PMO conjugated to the R_6_Gly peptide) trial revealed mean 11% of exon skipping and 5.7% of dystrophin expression after 7 monthly doses (Sarepta Therapeutics Announces Positive Data from Part B of MOMENTUM, a Phase 2 Study of SRP-5051 in Patients with Duchenne Muscular Dystrophy Amenable to Skipping Exon 51|Sarepta Therapeutics, Inc. https://investorrelations.sarepta.com/news-releases/news-release-details/sarepta-therapeutics-announces-positive-data-part-b-momentum). However, in November 2024, Sarepta announced the discontinuation of its vesleteplirsen programme on account of prolonged hypomagnesemia and a decline in the renal function marker eGFR (estimated glomerular filtration rate) in subsets of participants in the MOMENTUM trial (Community letter: update SRP-5051 program|Sarepta Therapeutics Available from https://investorrelations.sarepta.com/newsreleases/news-release-details/sarepta-therapeutics-announces-positive-data-part-b-momentum; Sarepta therapeutics announces third quarter 2024 financial results and recent corporate developments|Sarepta Therapeutics, Inc. Available from: https://investorrelations.sarepta.com/news-releases/news-release-details/sarepta-therapeutics-announces-third-quarter-2024-financial).

In the PepGen trial, a single PGN-EDO51 dose resulted in 2% of exon skipping in healthy volunteers (Larkindale et al. 2023). Interestingly, PepGen also observed transient hypomagnesemia in two PPMO-treated individuals, which resolved without intervention (PepGen reports positive data from phase 1 trial of PGN-EDO51 for the treatment of Duchenne muscular dystrophy|PepGen Available from: https://investors.pepgen.com/news-releases/news-release-details/pepgen-reports-positive-data-phase-1-trial-pgn-edo51-treatment). In contrast with the relatively simple amino acid composition of vesleteplirsen, the PGN-EDO51 is based on extensive iterative improvements of the Pip (PMO internalisation peptide) series of peptides developed through a collaboration between the groups of Mike Gait and Matthew Wood (Betts et al. 2012, 2015; Yin et al. 2011; Chwalenia et al. 2022). The latest versions of these peptides have been optimised to minimise renal toxicity without compromising exon skipping activity. It remains to be demonstrated whether PGN-EDO51 remains safe after prolonged treatment. Similarly, the PPMO compounds of Entrada are based on a cyclic peptide design, although more detailed chemical details of the structures of these compounds are not publicly available. Preliminary data from the phase 1 trial of ENTR-601-44 targeting *DMD* exon 44 (ENTR-601-44-101) in healthy volunteers suggested that the drug is safe, with exon skipping levels of up to 0.65% reported (Entrada therapeutics|Entrada therapeutics reports positive preliminary data in healthy volunteers from phase 1 ENTR-601-44-101 trial for Duchenne muscular dystrophy Available from: https://ir.entradatx.com/news-releases/news-release-details/entrada-therapeutics-reports-positive-preliminary-data-healthy). A phase 2 trial in amenable DMD patients is expected to be initiated in 2025.

Several PMO-bioconjugation strategies have been explored which target the ubiquitously-expressed transferrin receptor 1 (TFRC, Trf1), in order to promote uptake in skeletal and cardiac muscle tissues (Roberts et al. 2023). Avidity Biosciences is developing antibody oligonucleotide conjugates (AOCs) consisting of a monoclonal antibody targeting TFRC and a PMO designed to skip *DMD* exon 44 (i.e., Delpacibart zotadirseb, del-zota, or AOC 1044). This compound is currently under investigation in a phase 1/2 clinical trial (NCT05670730/EXPLORE44) with interim findings showing exon skipping levels of 1.5% after a single 10 mg/kg dose in healthy volunteers (Avidity biosciences reports positive data demonstrating AOC 1044 delivers unprecedented concentrations of PMO in muscle following a single dose in healthy volunteers from phase 1/2 EXPLORE44^TM^ trial for Duchenne muscular dystrophy Available from: https://www.prnewswire.com/news-releases/avidity-biosciences-reports-positive-data-demonstrating-aoc-1044-delivers-unprecedented-concentrations-of-pmo-in-muscle-following-a-single-dose-in-healthy-volunteers-from-phase-12-explore44-trial-for-duchenne-muscular-dystrophy-302013456.html).

Similarly, Dyne Therapeutics are developing DYNE-251, a Fab fragment targeting TFRC conjugated to a PMO designed to skip *DMD* exon 51. Dyne has recently reported positive preclinical findings from their platform technology (known as FORCE) (Desjardins et al. 2022). DYNE-251 is currently under investigation in a phase 1/2 clinical trial (NCT05524883/DELIVER) with interim data showing that patients treated at the 20 mg/kg dose exhibited 8.7% of healthy dystrophin levels (muscle content adjusted) and 3.7% (unadjusted) and functional improvements at 6 months post treatment for patients treated with both 20 mg/kg and 10 mg/kg monthly doses (Dyne reports positive phase 1/2 data for Duchenne agent DYNE-251 Available from: https://www.neurologylive.com/view/dyne-reports-positive-phase-1-2-data-duchenne-agent-dyne-251; Dyne therapeutics presents data at world muscle society congress highlighting promise of FORCE^TM^ platform to address underlying causes of neuromuscular diseases|Dyne Therapeutics, Inc. Available from: https://investors.dyne-tx.com/news-releases/news-release-details/dyne-therapeutics-presents-data-world-muscle-society-congress).

A discrepancy between RNA and protein levels has been a common observation across clinical programmes (Roshmi and Yokota 2023; Tillinger et al. 2023). For example, viltolarsen induced an 43.9% increase in exon-skipping which resulted only in production of 5.9% of the healthy dystrophin levels (Roshmi and Yokota 2023). It is well known that RNA and protein levels correlate poorly in general (Ideker et al. 2001; Roberts et al. 2015), although for genetic therapies a close relationship is often taken for granted. However, it is important to note that such inconsistences between levels of corrected transcripts and restored dystrophin protein may be a consequence of technical issues related to assay design, the timing of sample collection (i.e., there potentially being a lag between RNA level correction and the accumulation of dystrophin protein), differences in protein stability when comparing full-length healthy dystrophin with internally-deleted pseudodystrophins restored by exon skipping, or potentially other pathobiological features which may cause an obstacle to therapeutic success that are as yet under-appreciated (e.g., post-transcriptional repression of dystrophin expression via *trans*-acting factors in dystrophic muscle).

## Competition from microdystrophin gene therapy

Up until 2023, ASO-based drugs were the only option to treat the underlying cause of DMD. This changed with the approval of the delandistrogene moxeparvovec (elevidys, SRP-9001), an AAV-based gene replacement therapy developed by Sarepta Therapeutics (Hoy 2023). This drug encodes a minigene version of dystrophin, known as microdystrophin, in which large regions of the dystrophin open reading frame have been deleted in order for it to be small enough to be packaged into an AAV genome (Roberts et al. 2023). A phase 2 trial of elevidys demonstrated restoration of almost 40% of microdystrophin protein levels in patients at week 12 post-infusion (assessed by western blot) (Mendell et al. 2023). Despite relatively high levels of microdystrophin, improvements in clinical endpoints have so far been very modest. The strongest evidence for any benefit was in the 5–6 year age group, but the effect was much less apparent in older boys (Mendell et al. 2023). This raises the important question of whether microdystrophin(s), even when expressed at relatively high levels, are capable of replacing sufficient dystrophin function to allow meaningful clinical benefit.

Notably, in a parallel microdystrophin study sponsored by Genethon, investigators observed up to 85% of dystrophin positive myofibres by immunofluorescence 8-weeks post injection (First clinical trial results of gene therapy (GNT0004) for Duchenne muscular dystrophy presented at international myology (2024) Congress Available from: https://www.genethon.com/first-clinical-trial-results-of-gene-therapy-gnt0004-for-duchenne-muscular-dystrophy-presented-at-international-myology-2024-congress/). However, the gene replacement approach is likely to result in diminished pseudodystrophin production with time due to vector genome loss, epigenetic silencing of the transgene, and the dilution effect of non-transduced nuclei as a consequence of muscle growth and repair. Notably, repeat administration with AAV is currently challenging as the patient is effectively immunised to the treatment after the first dose (Mendell et al. 2023). Moreover, AAV poses a substantial safety risk with some clinical trials previously reporting fatal immune adverse effects using high doses of the viral vectors (including with the Pfizer microdystrophin) (High-dose AAV gene therapy deaths 2020). As such, the search for efficacious and safe DMD therapeutics is far from over, with ASOs, gene therapy, utrophin (a natural dystrophin paralogue) upregulation (Tinsley et al. 2011), and CRISPR-Cas9 (Tabebordbar et al. 2016; Hanson et al. 2022; Long et al. 2016) all showing promise (Roberts et al. 2023). While most of these strategies mimic dystrophin protein (gene replacement) and target the dystrophin mRNA (ASOs) or gene (CRISPR-Cas9), numerous aspects of dystrophin biology remain underexplored and poorly understood. For example, relatively little is known about the specific mechanisms governing correct dystrophin localisation to the sarcolemma. Our group has shown that the pattern of dystrophin coverage across the sarcolemma is an important consideration for effective restoration of muscle integrity (Westering et al. 2020), and that various therapeutic interventions restore dystrophin with distinct distribution patterns (Westering et al. 2020). Specifically, ASO-mediated exon skipping induced a uniform pattern of dystrophin distribution, whereas CRISPR-Cas9-mediated exon deletion resulted in a patchy pattern of dystrophin at the sarcolemma (Chwalenia et al. 2022; Hanson et al. 2022; Morin et al. 2023). As such, ASO therapy may present an advantage over other modalities in some cases, in terms of the uniformity of sarcolemmal dystrophin coverage post treatment.

## Conclusions

In conclusion, while great progress has been made in the field of ASO-mediated exon skipping therapies for DMD, there is still a need for better drug delivery and improved efficacy. These challenges may be addressed through the use of novel ASO chemical modifications and bioconjugation.

In this special *festschrift* issue, we recognise the career achievements of Professor Jenny Morgan. While Prof Morgan’s contributions to the field of satellite cell biology and myoblast transfer are widely recognised as world leading (Morgan et al. 1990, 1989; Watt et al. 1987; Partridge et al. 1989; Boldrin et al. 2015), she has also made key contributions to the field of ASO-mediated exon skipping. Prof Morgan was a founding member of the MDEX consortium, which was instrumental in the development of eteplirsen, and was a major contributor and co-author on the first studies to demonstrate dystrophin exon skipping in human DMD patient muscle (Kinali et al. 2009; Cirak et al. 2011). In addition, she has made numerous important contributions to this field, including development of a standardised method to assess exon-skipping in eteplirsen-treated patients and, more recently, in relation to the clinical evaluation of the exon 53 skipping compound golodirsen (Rossi et al. 2023; Anthony et al. 2012). An immortalised myoblast cell line derived from the *mdx* mouse (H2K-*mdx*) developed by Prof Morgan has become a widely-used cell model for screening exon skipping compounds that is still in use today (Morgan et al. 1994). Collectively, these contributions have been critical for the clinical realisation of exon skipping as a highly novel DMD therapeutic modality, and form the foundations on which future developments are built.

## Data Availability

No datasets were generated or analysed during the current study.

## References

[CR1] Aartsma-Rus A, Corey DR (2020) The 10th oligonucleotide therapy approved: Golodirsen for Duchenne muscular dystrophy. Nucleic Acid Ther 30:67–70 32043902 10.1089/nat.2020.0845 PMC7133412

[CR2] Aartsma-Rus A, Goemans N (2019) A sequel to the Eteplirsen Saga: Eteplirsen is approved in the United States but was not approved in Europe. Nucleic Acid Ther 29:13–15 30526286 10.1089/nat.2018.0756

[CR3] Aartsma-Rus A, Van Deutekom JCT, Fokkema IF et al (2006) Entries in the Leiden Duchenne muscular dystrophy mutation database: an overview of mutation types and paradoxical cases that confirm the reading-frame rule. Muscle Nerve 34:135–144 16770791 10.1002/mus.20586

[CR4] Aartsma-Rus A, Fokkema I, Verschuuren J et al (2009) Theoretic applicability of antisense-mediated exon skipping for Duchenne muscular dystrophy mutations. Hum Mutat 30:293–299 19156838 10.1002/humu.20918

[CR5] Aartsma-Rus A, Straub V, Hemmings R et al (2017) Development of exon skipping therapies for Duchenne muscular dystrophy: a critical review and a perspective on the outstanding issues. Nucleic Acid Ther 27:251–259 28796573 10.1089/nat.2017.0682 PMC5649120

[CR6] Amantana A, Iversen PL (2005) Pharmacokinetics and biodistribution of phosphorodiamidate morpholino antisense oligomers. Curr Opin Pharmacol 5:550–555 16087398 10.1016/j.coph.2005.07.001

[CR7] Anthony K, Feng L, Arechavala-Gomeza V et al (2012) Exon skipping quantification by quantitative reverse-transcription polymerase chain reaction in Duchenne muscular dystrophy patients treated with the antisense oligomer eteplirsen. Hum Gene Ther Methods 23:336–345 23075107 10.1089/hgtb.2012.117

[CR8] Avidity biosciences reports positive data demonstrating AOC 1044 delivers unprecedented concentrations of PMO in muscle following a single dose in healthy volunteers from phase 1/2 EXPLORE44^TM^ trial for Duchenne muscular dystrophy Available from: https://www.prnewswire.com/news-releases/avidity-biosciences-reports-positive-data-demonstrating-aoc-1044-delivers-unprecedented-concentrations-of-pmo-in-muscle-following-a-single-dose-in-healthy-volunteers-from-phase-12-explore44-trial-for-duchenne-muscular-dystrophy-302013456.html

[CR9] Betts C, Saleh AF, Arzumanov AA et al (2012) Pip6-PMO, a new generation of peptide-oligonucleotide conjugates with improved cardiac exon skipping activity for DMD treatment. Mol Ther Nucleic Acids 1:e38 23344180 10.1038/mtna.2012.30 PMC3438601

[CR10] Betts CA, Saleh AF, Carr CA et al (2015) Prevention of exercised induced cardiomyopathy following Pip-PMO treatment in dystrophic mdx mice. Sci Rep. 10.1038/srep08986 25758104 10.1038/srep08986 PMC4355666

[CR11] Bladen CL, Salgado D, Monges S et al (2015) The TREAT-NMD DMD Global Database: analysis of more than 7000 Duchenne muscular dystrophy mutations. Hum Mutat 36:395–402 25604253 10.1002/humu.22758 PMC4405042

[CR12] Boldrin L, Zammit PS, Morgan JE (2015) Satellite cells from dystrophic muscle retain regenerative capacity. Stem Cell Res 14:20 25460248 10.1016/j.scr.2014.10.007 PMC4305370

[CR13] Brad Wan W, Seth PP (2016) The medicinal chemistry of therapeutic oligonucleotides. J Med Chem 59:9645–9667 27434100 10.1021/acs.jmedchem.6b00551

[CR14] Bushby K, Finkel R, Birnkrant DJ et al (2010) Diagnosis and management of Duchenne muscular dystrophy, part 1: diagnosis, and pharmacological and psychosocial management. Lancet Neurol 9:77–93 19945913 10.1016/S1474-4422(09)70271-6

[CR15] Cardone N, Taglietti V, Baratto S et al (2023) Myopathologic trajectory in Duchenne muscular dystrophy (DMD) reveals lack of regeneration due to senescence in satellite cells. Acta Neuropathol Commun 11:1–11 37858263 10.1186/s40478-023-01657-z PMC10585739

[CR16] Chwalenia K, Oieni J, Zemła J et al (2022) Exon skipping induces uniform dystrophin rescue with dose-dependent restoration of serum miRNA biomarkers and muscle biophysical properties. Mol Ther Nucleic Acids 29:955–968 36159597 10.1016/j.omtn.2022.08.033 PMC9464767

[CR17] Cirak S, Arechavala-Gomeza V, Guglieri M et al (2011) Exon skipping and dystrophin restoration in patients with Duchenne muscular dystrophy after systemic phosphorodiamidate morpholino oligomer treatment: an open-label, phase 2, dose-escalation study. Lancet 378:595–605 21784508 10.1016/S0140-6736(11)60756-3 PMC3156980

[CR18] Clemens PR, Niizawa G, Feng J et al (2020a) The CINRG Becker natural history study: baseline characteristics. Muscle Nerve 62:369–376 32564389 10.1002/mus.27011 PMC7520226

[CR19] Clemens PR, Rao VK, Connolly AM et al (2020b) Safety, tolerability, and efficacy of viltolarsen in boys with Duchenne muscular dystrophy amenable to exon 53 skipping: a phase 2 randomized clinical trial. JAMA Neurol 77:982–991 32453377 10.1001/jamaneurol.2020.1264 PMC7251505

[CR20] Community letter: update SRP-5051 program|Sarepta therapeutics Available from: https://www.sarepta.com/community-letter-update-srp-5051-program

[CR21] Desjardins CA, Yao M, Hall J et al (2022) Enhanced exon skipping and prolonged dystrophin restoration achieved by TfR1-targeted delivery of antisense oligonucleotide using FORCE conjugation in mdx mice. Nucleic Acids Res 50:11401–11414 35944903 10.1093/nar/gkac641 PMC9723632

[CR22] Dhuri K, Bechtold C, Quijano E et al (2020) Antisense oligonucleotides: an emerging area in drug discovery and development. J Clin Med 9:1–24 10.3390/jcm9062004 PMC7355792 32604776

[CR23] Dyne reports positive phase 1/2 data for Duchenne agent DYNE-251 Available from: https://www.neurologylive.com/view/dyne-reports-positive-phase-1-2-data-duchenne-agent-dyne-251

[CR24] Dyne therapeutics presents data at world muscle society congress highlighting promise of FORCE^TM^ platform to address underlying causes of neuromuscular diseases|Dyne Therapeutics, Inc. Available from: https://investors.dyne-tx.com/news-releases/news-release-details/dyne-therapeutics-presents-data-world-muscle-society-congress

[CR25] England SB, Nicholson LVB, Johnson MA et al (1990) Very mild muscular dystrophy associated with the deletion of 46% of dystrophin. Nature 343:180–182 2404210 10.1038/343180a0

[CR26] Entrada therapeutics|Entrada therapeutics reports positive preliminary data in healthy volunteers from phase 1 ENTR-601-44-101 trial for Duchenne muscular dystrophy Available from: https://ir.entradatx.com/news-releases/news-release-details/entrada-therapeutics-reports-positive-preliminary-data-healthy

[CR27] First clinical trial results of gene therapy (GNT0004) for Duchenne muscular dystrophy presented at international myology (2024) Congress Available from: https://www.genethon.com/first-clinical-trial-results-of-gene-therapy-gnt0004-for-duchenne-muscular-dystrophy-presented-at-international-myology-2024-congress/

[CR28] Goemans N, Mercuri E, Belousova E et al (2018) A randomized placebo-controlled phase 3 trial of an antisense oligonucleotide, drisapersen, in Duchenne muscular dystrophy. Neuromuscul Disord 28:4–15 29203355 10.1016/j.nmd.2017.10.004

[CR29] Goyenvalle A, Griffith G, Babbs A et al (2015) Functional correction in mouse models of muscular dystrophy using exon-skipping tricyclo-DNA oligomers. Nat Med 21:270–275 25642938 10.1038/nm.3765

[CR30] Hanson B, Stenler S, Ahlskog N et al (2022) Non-uniform dystrophin re-expression after CRISPR-mediated exon excision in the dystrophin/utrophin double-knockout mouse model of DMD. Mol Ther Nucleic Acids 30:379–397 36420212 10.1016/j.omtn.2022.10.010 PMC9664411

[CR31] Heo Y-A (2020) Golodirsen: first approval. Drugs 80:329–333 32026421 10.1007/s40265-020-01267-2

[CR32] High-dose AAV gene therapy deaths 2020 High-dose AAV gene therapy deaths (2020) Nat Biotechnol 38: 910. 10.1038/s41587-020-0642-9 32760031

[CR33] Hoffman EP, Brown RH, Kunkel LM (1987) Dystrophin: The protein product of the Duchenne muscular dystrophy locus. Cell 51:919–928 3319190 10.1016/0092-8674(87)90579-4

[CR34] Hoy SM (2023) Delandistrogene moxeparvovec: first approval. Drugs 83:1323–1329 37566211 10.1007/s40265-023-01929-x

[CR35] Ideker T, Thorsson V, Ranish JA et al (2001) Integrated genomic and proteomic analyses of a systematically perturbed metabolic network. Science 292:929–934 11340206 10.1126/science.292.5518.929

[CR36] Ito K, Takakusa H, Kakuta M et al (2021) Renadirsen, a Novel 2’OMeRNA/ENA® Chimera antisense oligonucleotide, induces robust exon 45 skipping for dystrophin in vivo. Curr Issues Mol Biol 43:1267–1281 34698059 10.3390/cimb43030090 PMC8928966

[CR37] Kandasamy P, McClorey G, Shimizu M et al (2022a) Control of backbone chemistry and chirality boost oligonucleotide splice switching activity. Nucleic Acids Res 50:5443 35061895 10.1093/nar/gkac018 PMC9178015

[CR38] Kandasamy P, Liu Y, Aduda V et al (2022b) Impact of guanidine-containing backbone linkages on stereopure antisense oligonucleotides in the CNS. Nucleic Acids Res 50:5401–5423 35106589 10.1093/nar/gkac037 PMC9177980

[CR39] Kinali M, Arechavala-Gomeza V, Feng L et al (2009) Local restoration of dystrophin expression with the morpholino oligomer AVI-4658 in Duchenne muscular dystrophy: a single-blind, placebo-controlled, dose-escalation, proof-of-concept study. Lancet Neurol 8:918 19713152 10.1016/S1474-4422(09)70211-X PMC2755039

[CR40] Komaki H, Nagata T, Saito T et al (2018) Systemic administration of the antisense oligonucleotide NS-065/NCNP-01 for skipping of exon 53 in patients with Duchenne muscular dystrophy. Sci Transl Med. 10.1126/scitranslmed.aan0713 29669851 10.1126/scitranslmed.aan0713

[CR41] Larkindale J, Lonkar P, Goyal J et al (2023) P44 phase 1 study of PGN-EDO51 demonstrates tolerability, delivery and high levels of exon skipping for treatment of Duchenne muscular dystrophy (DMD). Neuromuscul Disord 33:S69

[CR42] Lauffer MC, van Roon-Mom W, Aartsma-Rus A (2024) Possibilities and limitations of antisense oligonucleotide therapies for the treatment of monogenic disorders. Commun Med 4:1–11 38182878 10.1038/s43856-023-00419-1 PMC10770028

[CR43] Lim KRQ, Maruyama R, Yokota T (2017) Eteplirsen in the treatment of Duchenne muscular dystrophy. Drug Des Devel Ther 11:533 28280301 10.2147/DDDT.S97635 PMC5338848

[CR44] Long C, Amoasii L, Mireault AA et al (2016) Postnatal genome editing partially restores dystrophin expression in a mouse model of muscular dystrophy. Science 351:400–403 26721683 10.1126/science.aad5725 PMC4760628

[CR45] Markati T, De Waele L, Schara-Schmidt U et al (2021) Lessons learned from discontinued clinical developments in Duchenne muscular dystrophy. Front Pharmacol. 10.3389/fphar.2021.735912 34790118 10.3389/fphar.2021.735912 PMC8591262

[CR46] McClorey G, Banerjee S (2018) Cell-penetrating peptides to enhance delivery of oligonucleotide-based therapeutics. Biomedicines 6:51 29734750 10.3390/biomedicines6020051 PMC6027240

[CR47] Mendell JR, Shieh PB, McDonald CM et al (2023) Expression of SRP-9001 dystrophin and stabilization of motor function up to 2 years post-treatment with delandistrogene moxeparvovec gene therapy in individuals with Duchenne muscular dystrophy. Front Cell Dev Biol. 10.3389/fcell.2023.1167762 37497476 10.3389/fcell.2023.1167762 PMC10366687

[CR48] Morgan JE, Coulton GR, Partridge TA (1989) Mdx muscle grafts retain the mdx phenotype in normal hosts. Muscle Nerve 12:401–409 2725568 10.1002/mus.880120511

[CR49] Morgan JE, Hoffman EP, Partridge TA (1990) Normal myogenic cells from newborn mice restore normal histology to degenerating muscles of the mdx mouse. J Cell Biol 111:2437 2277066 10.1083/jcb.111.6.2437 PMC2116381

[CR50] Morgan JE, Beauchamp JR, Pagel CN et al (1994) Myogenic cell lines derived from transgenic mice carrying a thermolabile T antigen: a model system for the derivation of tissue-specific and mutation-specific cell lines. Dev Biol 162:486–498 8150209 10.1006/dbio.1994.1103

[CR51] Morin A, Stantzou A, Petrova ON et al (2023) Dystrophin myonuclear domain restoration governs treatment efficacy in dystrophic muscle. Proc Natl Acad Sci USA 120:e2206324120 36595689 10.1073/pnas.2206324120 PMC9926233

[CR52] Muntoni F, Torelli S, Ferlini A (2003) Dystrophin and mutations: one gene, several proteins, multiple phenotypes. Lancet Neurol 2:731–740 14636778 10.1016/s1474-4422(03)00585-4

[CR53] Nicolau S, Malhotra J, Kaler M et al (2024) Increase in full-length dystrophin by exon skipping in Duchenne muscular dystrophy patients with single exon duplications: an open-label study. J Neuromuscul Dis. 10.3233/JND-230107 38461513 10.3233/JND-230107 PMC11091625

[CR54] Partridge TA, Morgan JE, Coulton GR et al (1989) Conversion of mdx myofibres from dystrophin-negative to -positive by injection of normal myoblasts. Nature 337:176–179 2643055 10.1038/337176a0

[CR55] Passamano L, Taglia A, Palladino A et al (2012) Improvement of survival in Duchenne muscular dystrophy: retrospective analysis of 835 patients. Acta Myologica 31:121 23097603 PMC3476854

[CR56] PepGen reports positive data from phase 1 trial of PGN-EDO51 for the treatment of Duchenne muscular dystrophy|PepGen Available from: https://investors.pepgen.com/news-releases/news-release-details/pepgen-reports-positive-data-phase-1-trial-pgn-edo51-treatment

[CR57] Petrof BJ, Shrager JB, Stedman HH et al (1993) Dystrophin protects the sarcolemma from stresses developed during muscle contraction. Proc Natl Acad Sci USA 90:3710 8475120 10.1073/pnas.90.8.3710 PMC46371

[CR58] Roberts TC, Johansson HJ, McClorey G et al (2015) Multi-level omics analysis in a murine model of dystrophin loss and therapeutic restoration. Hum Mol Genet 24:6756 26385637 10.1093/hmg/ddv381 PMC4634378

[CR59] Roberts TC, Langer R, Wood MJA (2020) Advances in oligonucleotide drug delivery. Nat Rev Drug Discovery 19:673–694 32782413 10.1038/s41573-020-0075-7 PMC7419031

[CR60] Roberts TC, Wood MJA, Davies KE (2023) Therapeutic approaches for Duchenne muscular dystrophy. Nat Rev Drug Discov 22:917–934 37652974 10.1038/s41573-023-00775-6

[CR61] Roshmi RR, Yokota T (2023) Viltolarsen: from preclinical studies to FDA approval. Methods Mol Biol 2587:31–41 36401022 10.1007/978-1-0716-2772-3_2

[CR62] Rossi R, Singh S, Torelli S et al (2023) P29 DMD transcript imbalance and nuclear trafficking evaluation in muscle biopsies from baseline and golodirsen treated 4053–101 clinical trial patients. Neuromuscul Disord 33:S104

[CR63] Sarepta therapeutics announces positive data from part B of MOMENTUM, a phase 2 study of SRP-5051 in patients with Duchenne muscular dystrophy amenable to skipping exon 51|Sarepta Therapeutics, Inc. Available from: https://investorrelations.sarepta.com/news-releases/news-release-details/sarepta-therapeutics-announces-positive-data-part-b-momentum

[CR64] Sarepta therapeutics announces third quarter 2024 financial results and recent corporate developments|Sarepta Therapeutics, Inc. Available from: https://investorrelations.sarepta.com/news-releases/news-release-details/sarepta-therapeutics-announces-third-quarter-2024-financial

[CR65] Servais L, Mercuri E, Straub V et al (2022) Long-term safety and efficacy data of Golodirsen in ambulatory patients with Duchenne muscular dystrophy amenable to exon 53 skipping: a first-in-human, multicenter, two-part, open-label, phase 1/2 trial. Nucleic Acid Ther 32:29 34788571 10.1089/nat.2021.0043 PMC8817703

[CR66] Shirley M (2021) Casimersen: first approval. Drugs 81:875–879 33861387 10.1007/s40265-021-01512-2

[CR67] Stephenson ML, Zamecnik PC (1978) Inhibition of Rous sarcoma viral RNA translation by a specific oligodeoxyribonucleotide. Proc Natl Acad Sci USA 75:285 75546 10.1073/pnas.75.1.285 PMC411231

[CR68] Summerton J, Weller D (1997) Morpholino antisense oligomers: design, preparation, and properties. Antisense Nucleic Acid Drug Dev 7:187–195 9212909 10.1089/oli.1.1997.7.187

[CR69] Szabo SM, Salhany RM, Deighton A et al (2021) The clinical course of Duchenne muscular dystrophy in the corticosteroid treatment era: a systematic literature review. Orphanet J Rare Dis 16:1–13 34022943 10.1186/s13023-021-01862-w PMC8141220

[CR70] Tabebordbar M, Zhu K, Cheng JKW et al (2016) In vivo gene editing in dystrophic mouse muscle and muscle stem cells. Science 351:407–411 26721686 10.1126/science.aad5177 PMC4924477

[CR71] Thada PK, Bhandari J, Umapathi KK (2023) Becker muscular dystrophy. StatPearls, St. Petersburg

[CR72] Tillinger M, Lake S, Servais L et al (2023) P22 WVE-N531 yields 53% mean exon 53 skipping in skeletal muscle of boys with Duchenne muscular dystrophy (DMD) after three biweekly doses. Neuromuscul Disord 33:S102

[CR73] Tinsley JM, Fairclough RJ, Storer R et al (2011) Daily treatment with SMTC1100, a novel small molecule utrophin upregulator, dramatically reduces the dystrophic symptoms in the mdx mouse. PLoS ONE 6:e19189 21573153 10.1371/journal.pone.0019189 PMC3089598

[CR74] Tsoumpra MK, Fukumoto S, Matsumoto T et al (2019) Peptide-conjugate antisense based splice-correction for Duchenne muscular dystrophy and other neuromuscular diseases. EBioMedicine 45:630–645 31257147 10.1016/j.ebiom.2019.06.036 PMC6642283

[CR75] van Westering TLE, Lomonosova Y, Coenen-Stass AML et al (2020) Uniform sarcolemmal dystrophin expression is required to prevent extracellular microRNA release and improve dystrophic pathology. J Cachexia Sarcopenia Muscle 11:578–593 31849191 10.1002/jcsm.12506 PMC7113513

[CR76] Vengalil S, Preethish-Kumar V, Polavarapu K et al (2017) Duchenne muscular dystrophy and Becker muscular dystrophy confirmed by multiplex ligation-dependent probe amplification: genotype-phenotype correlation in a large cohort. J Clin Neurol 13:91–97 28079318 10.3988/jcn.2017.13.1.91 PMC5242159

[CR77] Vincik LY, Dautel AD, Staples AA et al (2024) Evolving role of Viltolarsen for treatment of Duchenne muscular dystrophy. Adv Ther. 10.1007/s12325-024-02801-4 38376743 10.1007/s12325-024-02801-4

[CR78] Wagner K, Cripe L, Eagle M et al (2019) EP.83Design of a Phase 2/3 randomized controlled trial of suvodirsen (WVE-210201) in patients with Duchenne muscular dystrophy amenable to exon 51 skipping. Neuromuscul Disord 29:S176–S177

[CR79] Watt DJ, Morgan JE, Clifford MA et al (1987) The movement of muscle precursor cells between adjacent regenerating muscles in the mouse. Anat Embryol (Berl) 175:527–536 3578829 10.1007/BF00309688

[CR80] Wave life sciences announces positive interim data from FORWARD-53 Clinical Trial Evaluating WVE-N531 in boys with Duchenne muscular dystrophy amenable to exon 53 skipping - wave life sciences Available from: https://ir.wavelifesciences.com/news-releases/news-release-details/wave-life-sciences-announces-positive-interim-data-forward-53

[CR81] Wu B, Lu P, Benrashid E et al (2010) Dose-dependent restoration of dystrophin expression in cardiac muscle of dystrophic mice by systemically delivered morpholino. Gene Ther 17:132–140 19759562 10.1038/gt.2009.120

[CR82] Yin H, Saleh AF, Betts C et al (2011) Pip5 transduction peptides direct high efficiency oligonucleotide-mediated dystrophin exon skipping in heart and phenotypic correction in mdx mice. Mol Ther 19:1295–1303 21505427 10.1038/mt.2011.79 PMC3128823

[CR83] Zarrouki F, Relizani K, Bizot F et al (2022) Partial restoration of brain dystrophin and behavioral deficits by exon skipping in the muscular dystrophy X-linked (mdx) mouse. Ann Neurol 92:213–229 35587226 10.1002/ana.26409 PMC9544349

